# Exploring the sensitivities of experimental techniques to various types of membrane asymmetry using atomistic simulations

**DOI:** 10.1039/d4fd00200h

**Published:** 2025-01-16

**Authors:** Frederick A. Heberle, Milka Doktorova

**Affiliations:** a Department of Chemistry, University of Tennessee Knoxville Knoxville TN 37916 USA; b Department of Biochemistry and Biophysics, Stockholm University, Science for Life Laboratory Solna 171 65 Sweden milka.doktorova@dbb.su.se

## Abstract

Biological membranes have two leaflets that can differ in both lipid composition and total lipid abundance. These different types of asymmetries play a major role in determining the biophysical properties of the membrane; however, they have proven challenging to assay experimentally even in simpler model systems. Molecular dynamics simulations offer the means for detailed computational investigation of systematically varied interleaflet lipid distributions, but opportunities for critical validation with wet lab experiments are scarce. To help address this problem, here we use atomistic simulations of asymmetric bilayers to generate synthetic experimental data and thus investigate the sensitivity of various approaches to changes in relative lipid composition, number, and cholesterol distribution. Contrary to trends in symmetric bilayers, the simulations showed a decrease in lipid packing with increasing cholesterol in differentially stressed asymmetric bilayers, with more pronounced changes in the more loosely packed leaflet. Representative experimental data computed from the simulation trajectories indicated that the detection of asymmetry-induced changes in leaflet properties should be possible with environment-sensitive fluorescent probes and NMR observables, but may require optimization of sample preparation conditions. On the other hand, small-angle scattering data are already experimentally accessible and can reveal differential leaflet packing densities through a model-free analysis. We further show that computationally generated cryo-EM intensity profiles are highly sensitive to phospholipid imbalance between membrane leaflets. Together, these findings provide a roadmap for developing targeted applications of the *in vitro* techniques and obtaining experimental data critical for validating computationally derived principles related to membrane asymmetry.

## Abstract 2

We use atomistic simulations of lipid bilayers to generate representative datasets from model membranes *in vitro*, and examine the sensitivity of various experimental techniques to lipid abundance imbalances in compositionally asymmetric bilayers.

## Graphical abstract

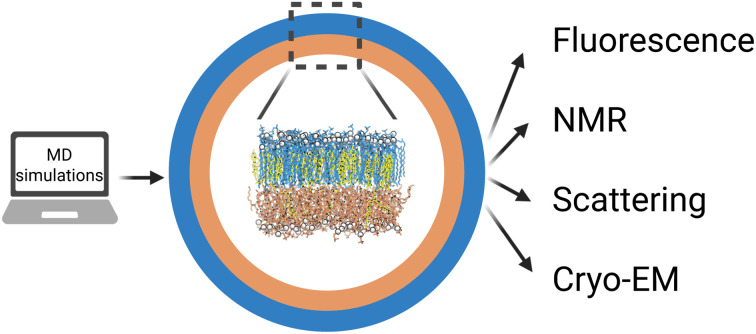

## Introduction

As biological interfaces, lipid bilayers have evolved to support a large number of functions and processes. This is achieved by a rich palette of biophysical properties created in part by the bilayer lipid composition.^1^ Extensive studies of the physio-chemical behavior of single component lipid bilayers have linked lipid structure (*e.g.*, chain length, head group type) to membrane permeability, fluidity and elasticity.^2–6^ Further analyses of the thermodynamics of lipid mixing have revealed the ability of membranes to compartmentalize their protein constituents *via* lateral heterogeneities characterized by in-plane (within leaflet) co-existence of regions with different lipid compositions and biophysical profiles.^7–9^ These versatile structure–property relationships have been investigated with a large suite of experimental and computational techniques,^10–13^ and formulate the basis for current models of membrane structure and function.

In addition to their in-plane complexity, lipid bilayers can exhibit non-random organization with respect to the two leaflets. For example, the existence and prevalence of compositional (and by extension biophysical) asymmetry in biological membranes has been recognized since the 1970s.^14–16^ Cells use valuable resources to actively maintain gradients in lipid saturation and charge across the bilayer midplane, which lead to contrasting fluidity and electrostatic surface potentials in the two leaflets.^16–20^ Lipids also have different spontaneous curvatures, and their interleaflet distributions may induce curvature stress in the bilayer.^2,21–23^ Cholesterol, with its unique structure and dynamics, further contributes to these effects *via* its (re)distribution.^21,24^ The overall compositional asymmetry of model and cell membranes has been assayed with various approaches including fluorescence quenching, peptide–membrane interactions, and NMR and mass spectrometry, although in many cases the exact quantification of the lipid make-up of both leaflets has not been possible.^16,25–29^

Lipid composition however, may not be the only (or even the main) determinant of the properties of a membrane. Lipid bilayers can tolerate a range of imbalances in the relative abundance of lipids between their leaflets.^22,30,31^ This is realized by adjustments in the lipid packing in each monolayer to conform to the area imposed by the closed membrane surface while minimizing hydrocarbon–water contacts. The result is a build-up of tension in one leaflet and pressure in the other (termed differential, or asymmetry, stress) with concomitant opposite effects on the structure and dynamics of the two leaflets.^32^ Analogously to externally applied net bilayer tension, differential stress can be tolerated up to a certain threshold before the membrane ruptures or changes morphology.^33^ For example, the addition of lipids to one leaflet of pre-formed membranes has been used to induce excessive asymmetry stress and budding or membrane shape changes in both synthetic and living systems.^34–37^ At the same time, theory and experiment indicate that substantial resting differential stress is likely present in giant synthetic vesicles with engineered compositional asymmetry as well as unperturbed cell plasma membranes, ‘silently’ affecting their measurable properties without any clear morphological signatures.^22,38–40^

Differential stress can be quantified from the lateral pressure distribution of simulated lipid bilayers.^22,30,41^ However, the ability to unambiguously detect and measure it in experiments remains a formidable challenge.^42^ To begin to address this problem, here we examine the sensitivities of various experimental techniques to different types of interleaflet membrane asymmetries including in phospholipid composition, number and cholesterol distribution. To this end, we employ molecular dynamics simulations to analyze the properties and dynamics of detailed atomistic models, then use them to generate representative experimental data from liposomes with the corresponding bilayer structures. The experiments we focus on include fluorescence lifetime measurements of environment-sensitive probes, NMR observables, small-angle scattering form factors, and cryo-EM images and intensity profiles. Our goal is to examine the differences in biophysical properties induced by the interplay of the various types of asymmetries and evaluate the capabilities of the *in vitro* approaches to detect them. Our results present a framework for identifying the conditions and controls for each technique that are best suited for analysis of the multifaceted asymmetry of lipid membranes, thus providing a roadmap to help guide future research efforts in these directions.

## Methods

### Atomistic bilayer models

Solvated lipid bilayer models were constructed with CHARMM-GUI^43–45^ and were composed of 1,2-dipalmitoyl-*sn*-glycero-3-phosphocholine (DPPC), 1,2-diarachidonoyl-*sn-glycero*-3-phosphocholine (DAPC) and cholesterol (Chol). In particular, eight symmetric bilayers had either DPPC or DAPC with 0, 10, 30 and 50 mol% Chol, and one symmetric bilayer (referred to as ‘scramble’) was composed of DPPC/DAPC/Chol 35/35/30 mol%. All bilayers contained 100 lipids per leaflet and were hydrated with 50 waters per lipid and no ions. Two additional symmetric bilayers with DPPC, 1,2-dioleoyl-*sn-glycero*-3-phosphocholine (DOPC) and Chol, were constructed, with DPPC/DOPC/Chol in molar percents of 56/9/35 (liquid-ordered, Lo) and 23/67/10 (liquid-disordered, Ld).

In addition, six asymmetric bilayers were taken from ref. 46 for further analysis. The bilayers had 70 DPPC lipids in one leaflet, a varying number of DAPC lipids in the opposite leaflet, and Chol at 30 mol% of all lipids distributed according to its chemical potential (as characterized from initial coarse-grained simulations, see ref. 46). Table 1 lists the number of lipid and cholesterol molecules in each leaflet. All bilayers were similarly constructed with CHARMM-GUI and hydrated with 50 waters per lipid and no ions.

**Table 1:** Composition and structure of simulated asymmetric bilayers. Shown are the name of each bilayer with the corresponding phospholipid imbalance (*N*_DPPC_/*N*_DAPC_ lipids in parentheses); the corresponding numbers of DPPC, DAPC and Chol molecules in each leaflet; the Chol leaflet mol%; area per lipid; leaflet thickness (distance between average *z* position of the phosphate atoms and bilayer center at *z* = 0); and tension (differential stress) in each leaflet with their corresponding errors in parenthesis

Bilayer (PL imb.)	Composition, # lipids (DPPC/DAPC/Chol)		Chol leaflet (mol%)		Area per lipid (Å^2^)		Leaflet thickness (Å), phosphate to midplane		Leaflet tension (mN m^−1^)	
	Top	Bot	Top	Bot	Top	Bot	Top	Bot	Top	Bot
A.1 (0.91)	70/0/56	0/77/7	44	8	42.0 (0.36)	63.0 (0.54)	23.5 (0.26)	20.2 (0.29)	14.8 (0.9)	−14.8 (0.9)
A.2 (1.00)	70/0/52	0/70/8	43	10	41.3 (0.30)	64.7 (0.46)	23.7 (0.22)	19.8 (0.28)	7.2 (0.9)	−7.2 (0.9)
A.3 (1.11)	70/0/47	0/63/10	40	14	40.8 (0.28)	65.4 (0.45)	23.9 (0.21)	19.5 (0.27)	−0.1 (1.0)	0.1 (1.0)
A.4 (1.25)	70/0/43	0/56/11	38	16	40.3 (0.19)	67.9 (0.33)	24.1 (0.18)	18.9 (0.26)	−11.1 (1.0)	11.1 (1.0)
A.5 (1.43)	70/0/37	0/49/14	35	22	40.1 (0.23)	68.1 (0.39)	24.1 (0.20)	18.7 (0.28)	−14.5 (1.0)	14.5 (1.0)
A.6 (2.00)	70/0/29	0/35/16	29	31	39.7 (0.20)	77.0 (0.39)	24.1 (0.17)	16.8 (0.28)	−31.7 (1.0)	31.7 (1.0)

### Molecular dynamics simulations

All bilayers were simulated with NAMD ^47^ and the CHARMM36 force field for lipids,^48^ as described in ref. 46. Briefly, the systems were first equilibrated with CHARMM-GUI’s 6-step equilibration protocol, then simulated with a 2 fs timestep and the length of all hydrogen bonds constrained (*rigidbonds* parameter set to *all*). Nonbonded interactions were modeled with a 10–12 Å Lennard-Jones potential function using force switching *via* the *vdwforceswitching* option in NAMD. The particle mesh Ewald method with a grid spacing of 1 Å was used for electrostatic interactions. The simulations were performed with semi-isotropic pressure coupling at a constant temperature of 40 °C (or 25 °C for the Lo and Ld bilayers) and pressure at 1 atm using Langevin thermostat (damping coefficient of 5 ps^−1^) and barostat (period 200 fs and decay 50 fs). The symmetric bilayers were run for 600–1180 ns. All asymmetric bilayers were run for over 1 µs with the last 400 ns used for analysis, as detailed in Table S4 in ref. 46.

### Calculation of bilayer structural parameters

Simulations were first centered so that the average position of all terminal methyl carbons was at (*x*,*y*,*z*) = (0,0,0) in every frame. Average area per lipid (APL) was calculated by dividing the lateral area of the simulation box by the number of lipids (including Chol) in each leaflet. Lateral pressure profiles were obtained with NAMD as described in detail in ref. 30. The differential stress and bilayer torque density were calculated from the integral and first moment of the pressure profiles, respectively, as outlined in ref. 46. The order parameters and relaxation rates of the carbon–hydrogen bonds were calculated following the protocols in ref. 49. Errors on APL and order parameters were obtained by block averaging, while errors on differential stress and bilayer torque density were calculated with bootstrapping analysis. Number and charge density profiles were computed for the whole systems in thin slabs of thickness 0.2 Å with the density profile plugin in VMD.^50^ The bilayer dipole potential was calculated from the charge density distribution following the approach in ref. 51.

### Calculation of small-angle scattering intensity

Scattering intensity was calculated from the time-averaged atomic number density distributions projected onto the bilayer normal, *n*_i_(*z*), where *z* denotes position along the bilayer normal and i indexes the individual lipid and water atoms. Each distribution was scaled by the appropriate atomic scattering factor (*i.e.*, the X-ray atomic form factor or the coherent neutron scattering length) and then summed to produce a scattering length density profile, *ρ*(*z*). The scattering intensity as a function of scattering vector *q* is given by:^52^1
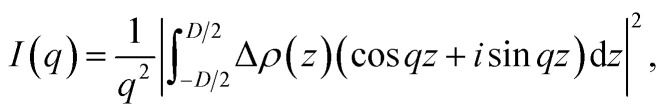
where Δ*ρ*(*z*) = *ρ*(*z*) − *ρ*_s_ with *ρ*_s_ being the scattering length density of the aqueous solvent, and the integral extends from the bulk solvent (*i.e.*, where Δ*ρ*(*z*) = 0) on one side of the bilayer to the other (*D* is the length of the simulation box). The scattering form factor was then computed from the intensity profile as 
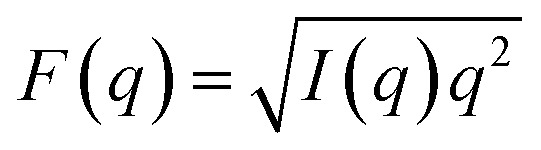
. The center-of-mass of the form factor was calculated as:2a
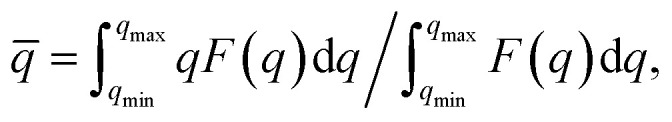
2b
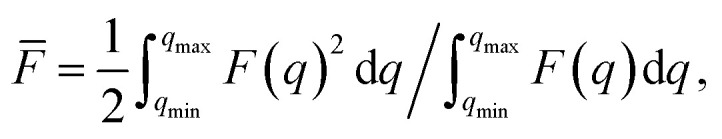
where the *q*-range (*q*_min_ to *q*_max_) was 0.03–0.37 Å^−1^ for SAXS data and 0–0.30 Å^−1^ for SANS data.

### Calculation of cryo-EM intensity profiles

In a projection image of a liposome, the azimuthally averaged intensity profile (IP) contains information about the bilayer structure. IPs were calculated as previously described^53^ and briefly summarized here. First, the electrostatic potential within the lipid bilayer, *Φ*(*z*), was approximated as a sum of contributions from individual lipid and water atoms with an additional contribution from the membrane dipole potential *Φ*_d_, *i.e.*,3
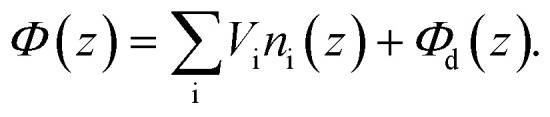
In eqn (3), the sum is over individual lipid atoms, *n*_i_(*z*) is the atom’s number density profile described in the previous section, and *V*_i_ is the spatially integrated, shielded coulomb potential for an isolated neutral atom (*V*_i_ = 25, 130, 108, 97, and 267 V Å^3^ for H, C, N, O and P, respectively). The phase shift experienced by an electron wave passing through the bilayer is given by:

4 *g*(*z*) = *σ*_e_*Φ*(*z*),

where *σ*_e_ accounts for the dependence of the electron phase on the projected potential and is equal to 0.65 mrad V^−1^ Å^−1^ for 300 keV electrons.^54^ A cryo-EM image of a liposome corresponds to a projection of the vesicle’s spherical scattering profile, *γ*(*r*,*θ*,*ϕ*), onto a plane, followed by convolution with a contrast transfer function (CTF). For a spherically symmetric liposome of radius *R* where each leaflet is uniformly mixed, *γ* has no angular dependence and can be approximated with the flat bilayer profile *g*(*z*) using the coordinate transformation *r* = *z* + *R*, such that *γ*(*r*,*θ*,*ϕ*) = *γ*(*r*) = *g*(*r* − *R*). In this case, the projected density *Γ*(***r***) is given by the Abel transform:5
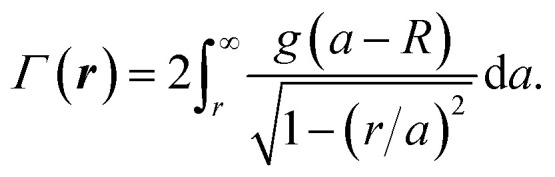


To mimic experimental images, *Γ*(***r***) is convolved with a CTF, *c*(***s***), and associated phase perturbation factor, *χ*(***s***):

6 *c*(***s***) = [sin *χ*(***s***) − *Q* cos *χ*(***s***)]exp(−*B*|***s***|^2^),

7
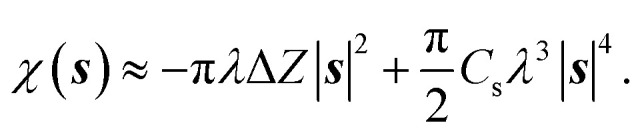
In eqn (6) and (7), ***s*** is the spatial frequency, *B* is the amplitude decay factor, *Q* is the dimensionless amplitude contrast factor, *λ* is the electron wavelength, Δ*Z* is the defocus length (defined such that positive values indicate underfocus), and *C*_s_ is the spherical aberration coefficient. The reciprocal space image is calculated as:8
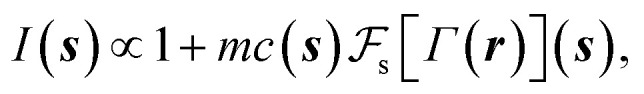
where 
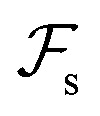
 is the Fourier transform of the projected 2D phase shift image *Γ*(***r***) and *m* is an arbitrary scale factor that adjusts the intensity contrast in the spatial domain. The corresponding real-space image *I*(***r***) is the inverse Fourier transform of *I*(***s***),9
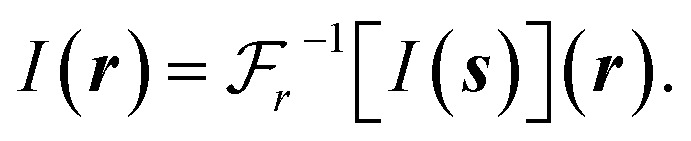


For the calculations in this work, we used the following cryo-EM parameter values: *R* = 50 nm, *B* = 300 Å^2^, *Q* = 0.075, *λ* = 1.97 pm (corresponding to 300 keV electrons), Δ*Z* = 2.5 µm, *C*_s_ = 2.0 mm, *m* = 400.

## Results and discussion

### Asymmetry-mediated changes in leaflet composition and properties

To investigate the effects of biologically relevant asymmetries on membrane properties, we analyzed a series of molecular dynamics (MD) simulation trajectories of all-atom bilayers where one leaflet contained the saturated lipid DPPC, the other contained the unsaturated lipid DAPC, and cholesterol was at a fixed overall concentration and free to redistribute between the leaflets. This simplified lipid composition aims to capture the asymmetry in lipid saturation reported for red blood cell membranes,^16^ which also plays a key role in determining the chemical potential of cholesterol in the leaflets.^24^ The bilayers had a fixed number of DPPC lipids (*N*_DPPC_) in the top leaflet but varying numbers of DAPC lipids (*N*_DAPC_) in the bottom leaflet (Table 1). Initial coarse-grained simulations showed that while such bilayers with more DPPC than DAPC lipids were stable up to relatively large imbalances, deviations in the opposite direction (more DAPC lipids) were not tolerated as well and produced unphysical bilayer morphologies even at smaller imbalances.^46^ The phospholipid (PL) imbalance, *N*_DPPC_/*N*_DAPC_, in the systems we studied here thus ranged from 0.91 to 2.0.

**Fig. 1:**
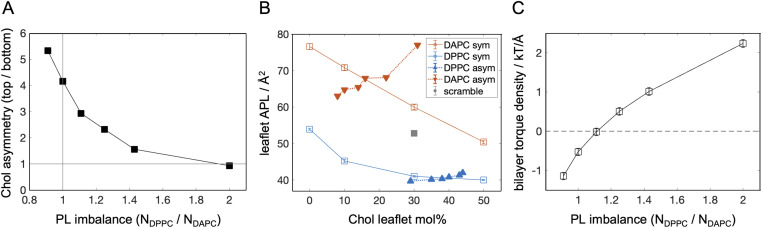
Biophysical properties of simulated asymmetric bilayers. (A) Cholesterol asymmetry (cholesterol mol% in the top *vs.* the bottom leaflet) as a function of interleaflet PL imbalance. Solid gray lines indicate the conditions of no PL imbalance and no cholesterol asymmetry. (B) Leaflet area per lipid (APL) as a function of cholesterol mol% in the individual leaflets of symmetric DPPC or DAPC bilayers (open squares, ‘*sym*’) and the asymmetric DPPC/DAPC bilayers from Table 1 (filled triangles, ‘*asym*’). Shown for comparison is a symmetric ‘scramble’ bilayer with 1 : 1 DPPC : DAPC and 30 mol% cholesterol (gray square). (C) Bilayer torque density as a function of PL imbalance in the asymmetric bilayers from Table 1.

All systems had cholesterol at 30 mol% of all PLs. In real membranes, cholesterol can quickly flip between leaflets and redistribute according to its chemical potential. Since proper sampling of this movement is challenging in all-atom representation, the equilibrium cholesterol distribution in the models was determined from initial long coarse-grained trajectories, and did not change during the all-atom simulations (Table 1).^46^ In all but one of the bilayers, cholesterol showed a strong preference for the saturated DPPC leaflet (Fig. 1A). Its strong pairwise interactions with DPPC competed with other factors including the overabundance of the saturated lipid (relative to DAPC), as predicted from theory.^24^ Cholesterol’s enrichment in the top leaflet thus decreased gradually across the systems until it completely disappeared in the A.6 bilayer which had twice as many DPPC than DAPC lipids (Fig. 1A and Table 1).

Cholesterol has a well characterized condensing effect on lipid packing in both saturated and unsaturated membranes.^55–57^ We confirmed this trend in symmetric DPPC and DAPC bilayers which showed a systematic decrease in the average area per lipid (APL) with increasing amounts of cholesterol (Fig. 1B, open symbols). Since the individual leaflets of the asymmetric bilayers are binary mixtures with varying concentrations of cholesterol, we can similarly analyze the effect of the sterol on their APLs. In contrast to the expected trend observed in the symmetric membranes, cholesterol had an exactly opposite effect in the asymmetric bilayers, increasing rather than decreasing the leaflet APL (Fig. 1B, closed symbols). This counter-intuitive trend can be related to the internal stresses in the leaflets, *i.e.* differential stress, resulting from the interleaflet PL and cholesterol distributions (Table 1). In most bilayers, one leaflet had tension while the lipids in the other leaflet were compressed (*i.e.*, a negative tension). These stresses constrained the effects of cholesterol, making them deviate from the trends observed in symmetric bilayers which by definition have zero leaflet tension.^30^ Only the A.3 bilayer, which had a PL imbalance of 1.11 and about 3-fold more cholesterol in the DPPC leaflet, had no differential stress. Interestingly, in that bilayer, the APL of the DAPC leaflet containing 14 mol% cholesterol (Table 1) was lower than the APL of a symmetric DAPC bilayer with similar cholesterol concentration, while the condensing effect of cholesterol in the DPPC leaflet was similar to its symmetric counterpart (Fig. 1B). These results reveal that even in the absence of internal stresses, compositional asymmetry can influence bilayer properties in ways that cannot be fully recapitulated in symmetric bilayers, which is a manifestation of interleaflet coupling.^58–60^ How the specific lipid compositions of the leaflets affect the observed relationships between PL imbalance and differential stress, and APL and cholesterol concentration, remains to be investigated.

The high resolution of the simulated bilayers also enables analysis of the propensity of the bilayers to curve, which in experiments can be monitored *via* changes in membrane morphology.^34,61,62^ Depending on its lipid composition, each leaflet has a spontaneous curvature that determines the curvature stress experienced by the leaflet in a given configuration (*e.g.*, when flat).^63,64^ The balance between curvature stress and differential stress governs the energy cost of changes in the bilayer morphology.^22^ In particular, the first moment of the lateral pressure profile (whose integral gives the leaflet stresses) is the bilayer torque density.^41^ Its deviation from zero provides an estimate of the bilayer tendency to curve, with positive and negative values indicating curvature towards the top and bottom leaflets, respectively. Analysis of the asymmetric bilayers shows that this parameter is highly sensitive to their molecular organization, with a consistent preference for curvature towards the compressed leaflet (Fig. 1C). Only in the tensionless A.3 bilayer is a flat morphology the most energetically favorable. Since this bilayer has no area strain, the result implies that it has vanishing curvature stress (*i.e.*, a spontaneous curvature of zero)^41^ despite its highly asymmetric profile (Table 1). This is likely due to the modulation of the leaflets’ spontaneous curvature by cholesterol.^21^ However, vanishing differential stress is not a pre-requisite for a stable flat configuration, as the latter can exist even when the leaflets experience significant tension and compression that counterbalances curvature stress.^46^ These findings demonstrate that the absence of curvature in experimental asymmetric membranes cannot be used to infer the presence or absence of both differential and curvature stresses.

### Sensitivity of environment-sensitive probes

**Fig. 2:**
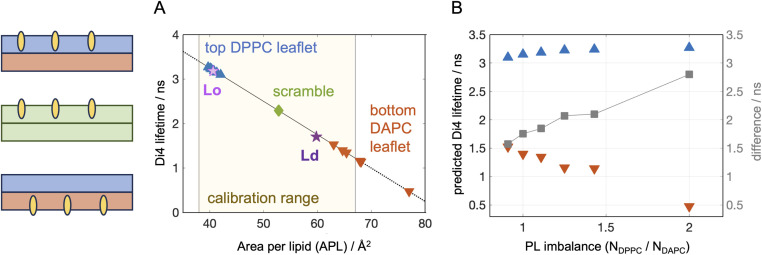
Predicted sensitivity of Di4 lifetime to membrane asymmetries. (A) Fluorescence lifetime of Di4 calculated from leaflet APLs in simulated bilayers using the experimentally derived calibration curve from ref. 46. Shown is data for the simulated top DPPC leaflets (blue triangles pointing up), bottom DAPC leaflets (red triangles pointing down) and symmetric scrambled bilayer (green diamond). Also shown are the results for symmetric 3-component Lo and Ld compositions of DPPC/DOPC/Chol as explained in the text (pink and purple stars). The solid black curve shows the linear relationship between simulation and experiment derived in ref. 46 from model membranes, while the dotted lines indicate regions outside of the calibration range. (B) Calculated Di4 lifetime as a function of PL imbalance for the top and bottom leaflets in the asymmetric bilayers (same symbols as in (A)). Also shown is the difference between the two (gray squares, right *y*-axis) which shows the largest sensitivity to the transverse lipid organization in the membranes.

One commonly applied approach for analyzing the structural properties of lipid membranes in both model systems and cells, is measuring changes in their lipid packing *via* environment-sensitive probes.^65^ A typical experiment relies on incorporation of the probe in one or both bilayer leaflets and recording its fluorescence lifetime (for *e.g.* Di4-ANEPPDHQ,^66^ Flipper^67^) or generalized polarization (laurdan,^68^ NR12S ^69^). While each of these probes likely senses an interplay of multiple leaflet properties,^70^ they are often used as reporters of lipid packing. This has been shown to be a good approximation for Di4, whose lifetime in symmetric giant unilamellar vesicles is very strongly correlated with the average area per lipid calculated from corresponding simulations of flat bilayer patches for a wide range of simple 1- and 2-component lipid mixtures.^46^ To verify that the relationship holds for 3-component bilayers, we simulated the two timeline endpoints of DPPC/DOPC/Chol 40/40/20 representing its coexisting Lo and Ld compositions (see Methods). The bilayers were thus symmetric and uniform, and we compared the APLs from the simulations to the experimentally measured Di4 lifetimes in the mixtures from ref. 16. To account for any differences in the experimental conditions between ref. 16 and 46, we used the Di4 lifetime of DOPC reported in both studies to normalize the results. The Lo and Ld compositions both fell precisely on the calibration curve as shown in Fig. 2A, confirming that even in more complex multi-component mixtures, Di4 lifetime is highly correlated with simulated APLs.

Using the experimentally derived linear relationship between the two parameters, we then converted the leaflet APLs in our other simulated bilayers to Di4 lifetimes and compared the sensitivity of this parameter to the asymmetries in the membranes (Fig. 2). Since Di4 is charged and does not easily flip between leaflets,^16^ we analyzed the expected lifetime measured when the probe is added to the bilayer from the outside, labeling only the top leaflet, or selectively incorporated exclusively in the bottom leaflet. Thus, while the simulations do not contain the probe itself, the structural properties of the leaflets are used to estimate the leaflet-specific fluorescence lifetime read-out from Di4 in an experiment. In experiments, adding Di4 to outer membrane leaflets is straightforward since in aqueous solutions the probe partitions strongly into lipid bilayers due to its hydrophobic groups. Adding Di4 to selectively label the inner (cytosolic) leaflet is more challenging but has been accomplished experimentally by microinjection of cells.^16^ This was confirmed by the difference in Di4 lifetime of the cytoplasmic leaflet relative to the exoplasmic leaflet, and the lack of BSA back-extraction from the PM (see Fig. S7 in ref. 16). In model membranes, selective labeling of the inner leaflet of liposomes has been accomplished using cyclodextrin to exchange the outer leaflet probe with lipid from unlabelled donor vesicles,^71^ although this has not been specifically demonstrated for Di4. The selective incorporation of a lipid dye in the inner leaflet can be verified with externally added quenchers, and other environment-sensitive probes can in principle be used in analogous ways (see Introduction).

The first noticeable result from our computational analysis was that two of the data points—corresponding to two DAPC-rich leaflets—fell outside of the calibration range between simulation and experiment (Fig. 2A).^46^ One of them is a leaflet in bilayer A.6 that has the most extreme PL imbalance of 2.0 and substantial differential stress (Table 1) and bilayer torque density (Fig. 1C). This highly stressed bilayer state is tolerated in simulations due to the relatively small system size and applied periodic boundary conditions but may exceed the tolerance threshold of liposomes *in vitro*, where the stress can be relieved instead by mechanisms such as extreme curvature and budding.^34,72^ The calibration range could be extended by analyzing more disordered symmetric bilayers under net bilayer tension or at higher temperatures (both of which can be applied in simulations and experiments to increase leaflet APL). However, the tolerance threshold for bilayer stress in the experiments, as well as any potential changes to the correspondence between the simulations and Di4 measurements from external perturbations, must be investigated and accounted for.

Comparison between the calculated Di4 lifetimes of the outer and inner leaflets across the series of simulated bilayers reveals differences in the sensitivity of this parameter to the properties of the respective lipid compositions (Fig. 2B). The relatively tightly packed DPPC leaflet, whose APL varies only from 39.7 ± 0.2 Å^2^ to 42 ± 0.36 Å^2^ between the two extremes (bilayers A.1 and A.6), shows marginal variation in Di4 lifetime. In contrast, the predicted changes in the DAPC leaflet, whose APL increases by 22% (Table 1), are more pronounced. Since the trends in the leaflet APLs are inversely correlated, where the smaller APL becomes smaller while the larger APL increases, most sensitive to the asymmetries is the difference between outer and inner leaflet Di4 lifetimes (Fig. 2B, gray squares). Measuring this difference *in vitro* would require concurrent experiments of membranes with either their top or bottom leaflets labelled with Di4, or calibrating and incorporating in the two leaflets different environment-sensitive probes with non-overlapping fluorescence spectra that can be imaged and analyzed simultaneously.

### Sensitivity of NMR structural parameters

**Fig. 3:**
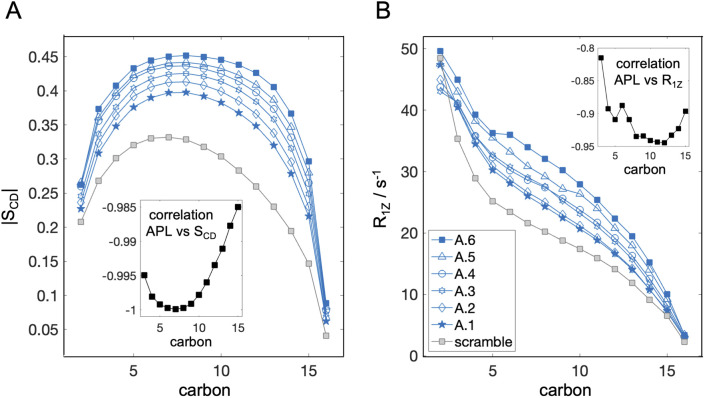
Variation in simulation parameters corresponding to properties measured with NMR. (A) Acyl chain order parameter profiles for carbons 2 through 16 averaged over the *sn*-1 and *sn*-2 chains of DPPC in the asymmetric bilayers A.1 to A.6 from Table 1, and the scramble analog with DPPC/DAPC 1 : 1 and 30 mol% cholesterol. Errors are less than 10^−2^ and are omitted for clarity. (B) Spin-lattice relaxation rates of the corresponding CH bond fluctuations analyzed in (A). Insets in both (A) and (B) show the correlation between *S*_CD_ or *R*_1Z_ and leaflet APL (Table 1) calculated from the plotted data for the asymmetric bilayers.

In addition to APL, bilayer structure is often characterized by the degree of order of the lipid acyl chains. This property is quantified from the dynamic reorientation of the carbon–hydrogen (CH) bonds along the lipid chains relative to the bilayer normal. In particular, the average order parameter (*S*_CD_) characterizing the mean of the fluctuations, as well as their relaxation rate (*R*_1Z_), can be measured with NMR and also calculated from simulations (*e.g.* ref. 49 and 73). The experiments are often done with deuterium NMR which allows for better spectral resolution and identification of chain segments.^74^ The measurements are typically performed on oriented lipid stacks or multilamellar lipid dispersions,^75^ but can also be done on unilamellar liposomes,^76,77^ thus making them suitable for asymmetry studies using model membranes. Since deuterated lipids with saturated chains are in general more readily available from commercial vendors, we analyzed the CH bond dynamics for DPPC in the simulated bilayers as representative experimental data (Fig. 3). Similar comparisons of the order at specific carbon segments can also be made with electron spin resonance (ESR) experiments *via* spin probes selectively incorporated into one of the leaflets.^71,78^

The resulting order parameter profiles show an overall increase in chain order (*i.e.*, higher |*S*_CD_|) with a decrease in leaflet tension and APL (Fig. 3A and Table 1), as expected. There is a strong negative correlation between APL and *S*_CD_ for all carbons (inset in Fig. 3A) but the best sensitivity to the asymmetry-mediated properties (*i.e.*, the largest separation between all conditions) is achieved in the bottom parts of the chains, at carbons 12 through 15 (Fig. 3A). This is not surprising since this is the part of the leaflet in closest contact with the opposing leaflet. In contrast, the order parameter profiles for some bilayers (*e.g.* A.5 and A.4) overlap in the plateau region (carbons 5 through 10). Interestingly, in the same bilayers, these carbon segments have detectable differences in their relaxation rates even though the correlation between APL and *R*_1Z_ is slightly weaker (Fig. 3B), indicating that the structural information from *S*_CD_ and *R*_1Z_ is complementary. Thus, if available, data from both parameters can maximize the ability to distinguish the leaflet states in experiments. Most challenging in this endeavour would be optimizing the size and hydration level of the asymmetric vesicles for the measurements. While the simulations revealed overall strong correlation between the NMR parameters and APL (Fig. 3), experimental conditions may be key for determining the consistency of the measured bilayer structure with other techniques.^73^ The relationship between the order parameters and relaxation rates in the region of leaflet–leaflet interaction has also been connected to bilayer elasticity *via* the membrane thickness in symmetric membranes.^49^ Further research is needed to test and develop this dependence in asymmetric bilayers.

### Sensitivity of small-angle scattering data

**Fig. 4:**
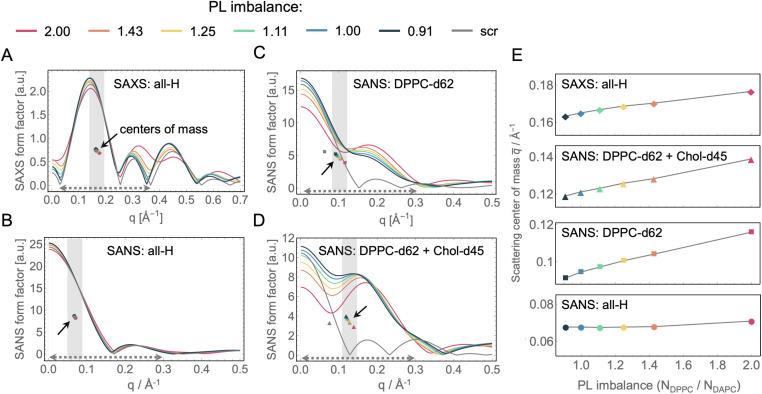
Small-angle scattering data from simulated bilayers. (A) Small-angle X-ray scattering (SAXS) form factors for the asymmetric and scramble bilayers calculated from the simulation trajectories as a function of *q*. Also shown are the corresponding center-of-mass for each scattering form factor (filled symbols) as explained in the text. (B–D) Small-angle neutron scattering (SANS) form factors for the simulated bilayers for three isotopic conditions: fully protiated (hydrogenated) sample with either none (all-H), only DPPC chains (DPPC-d62) or both DPPC chains and Chol (DPPC-d62 + Chol-d45) perdeuterated. In all samples, water molecules were 100% deuterated. The corresponding scattering centers-of-mass are also shown (filled symbols). (E) Plots of the *q*-values of the scattering centers-of-mass as a function of PL imbalance for each of the data sets in A–D. All plots span *q*-regions of the same width (*i.e.*, they have equivalent *y*-axis ranges) as illustrated by the gray shaded areas on the plots in A–D. Note that these regions are different from the *q*-ranges used to calculate the centers-of-mass which are indicated as dotted arrows near the horizontal axis in panels A–D.

Small-angle X-ray and neutron scattering (SAXS and SANS, respectively) are robust and well-established techniques for the precise measurement of bilayer structural parameters including area per lipid and bilayer thickness.^79,80^ Using atomic number density profiles from simulated bilayers as input, we calculated form factors for SAXS (Fig. 4A) and SANS (Fig. 4B–D), the latter using different combinations of protiated and perdeuterated lipids to explore the effects of internal bilayer contrast. Whereas symmetric bilayers produce clearly delineated scattering lobes separated by minima that approach zero intensity, asymmetry results in a characteristic “liftoff” of the minima that is particularly pronounced in cases of extreme transbilayer scattering length density contrast (*e.g.*, protiated DAPC in the inner leaflet and DPPC-d62 in the outer leaflet as in Fig. 4C and D).^81–83^ Such liftoff is also visible (albeit to a smaller extent) in the first minimum following the first peak at the lowest *q* in the SAXS form factors (Fig. 4A). In principle, structural parameters can be extracted from the data by fitting to a suitable structural model; however, this is a daunting task for an asymmetric membrane where each leaflet has its own variables thus requiring a multitude of free parameters in the fitting routine.^84,85^ Furthermore, as illustrated in the fully protiated condition in Fig. 4B, bilayers with very different PL and cholesterol asymmetries can lead to almost indistinguishable scattering curves, making it difficult to unambiguously determine the underlying lipid organization.

An alternative approach to analyzing such data uses model-free quantities that correlate with structural features. For example, increasing phospholipid imbalance results in systematic changes in the intensity profiles that are particularly prominent in the first two scattering lobes for SAXS (Fig. 4A) and internally contrasted SANS (Fig. 4C and D) data. These changes are readily apparent in the centers-of-mass of the *F*(*q*) curves, calculated as described in the Methods and plotted as small filled symbols (highlighted by solid arrows) in Fig. 4A–D. Fig. 4E shows the corresponding trends and reveals that the *q*-coordinate of the center-of-mass increases nearly linearly with phospholipid imbalance in all but the fully protiated SANS data. A useful aspect of this analysis is that it is insensitive to sample-to-sample differences in lipid concentration. Furthermore, the *q*-range over which it is calculated can be adjusted to include only data with the highest signal-to-noise in the experiments. Importantly, this quantification demonstrates that both SAXS and SANS with at least one asymmetrically distributed perdeuterated lipid are sensitive to compositional and number imbalances, in contrast to SANS of fully protiated samples. Joint analysis of these various conditions may thus provide the best approach for identifying a bilayer model with the correct structure.

### Sensitivity of cryo-EM imaging and analysis

**Fig. 5:**
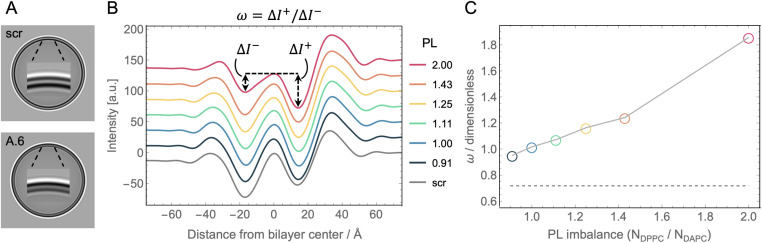
Cryo-EM intensity profiles calculated from simulation trajectories. (A) Synthetic cryo-EM images of 100 nm-diameter vesicles corresponding to the scramble (top) and asymmetric A.6 (bottom, see Table 1) simulated bilayers. Shown in the middle of each vesicle is an enlargement of the intensity close to and across the bilayer, showing how the contrast between the outer and inner troughs in the two images is reversed, consistent with the change in PL imbalance in the bilayer leaflets. (B) Intensity profiles for the asymmetric bilayers (color-coded according to their PL imbalance), and the scrambled bilayer (gray) computationally derived from the simulated atomic number density profiles and membrane dipole potential as described in Methods. The outer (Δ*I*^+^) and inner (Δ*I*^−^) trough depths relative to the central peak which define the parameter *ω*, are illustrated for the curve corresponding to a PL imbalance of 2.0. Profiles have been shifted vertically for better visualization. (C) *ω* as a function of PL imbalance for all asymmetric bilayers (symbols). *ω* for the scramble membrane is also shown for comparison (dashed line).

Cryo-EM enables the direct visualization of lipid bilayers at sub-nanometer resolution.^86,87^ When spherical liposomes are imaged in projection, the defining characteristic is a pair of dark concentric circles roughly corresponding to the electron dense lipid headgroup layers (Fig. 5A). For laterally homogeneous bilayers, images of individual vesicles can be reduced to 1D intensity profiles normal to the bilayer by azimuthal integration and then ensemble averaged to produce data suitable for quantitative analysis.^88,89^ As described in Methods, the cryo-EM image formation process for liposomes can also be accurately mimicked computationally using atomic number density profiles from MD simulations as input.^90^Fig. 5B shows cryo-EM intensity profiles calculated from the simulated asymmetric bilayers with varying phospholipid imbalance, as well as the symmetric scrambled bilayer with 30 mol% Chol.

Because the intensity profiles arise from electron density variation across the bilayer, their features are sensitive to details of the bilayer structure. For example, the distance between the two deep troughs has been shown to be strongly correlated with bilayer thickness in both simulated and experimental data.^91^ Similarly, the depth of the troughs can reveal differences in lipid packing density within the leaflets.^53^ To explore this further, we defined a model-free parameter, *ω* = Δ*I*^+^/Δ*I*^−^, calculated as the ratio of the outer (Δ*I*^+^) and inner (Δ*I*^−^) trough depths relative to the central peak as depicted in Fig. 5B. Fig. 5C shows that *ω* nearly doubled (from 0.95 to 1.86) when the phospholipid imbalance was varied from 0.9 to 2.0. It is important to note that *ω* for perfectly symmetric bilayers is not unity but instead ranges from ∼0.65–0.8 (*i.e.*, the outer trough is shallower than the inner trough) due to the spherical geometry of the liposome and the nature of projection imaging, as shown by the scrambled bilayer in Fig. 5B (gray curve). These results demonstrate that a simple model-free analysis can be used to accurately estimate the phospholipid imbalance of liposomes, provided that a sufficient number of liposomes are analyzed to achieve high signal-to-noise in the intensity profile. Whether the trends in *ω* for asymmetric bilayers can be affected by variations in lipid composition, cholesterol concentration and electron contrast-enhancers (such as substitutions of hydrogen in the lipid molecules with halogen atoms) needs to be further explored.

## Conclusion

While the compositional asymmetry of biological membranes has been known for over half a century, it has only recently been recognized that an asymmetric membrane cannot solely be defined as just having leaflets with different lipid compositions. Imbalances in the total lipid abundances of the two monolayers can accompany interleaflet compositional variations and have profound effects on the bilayer biophysical properties. This makes the detection and characterization of both compositional and number asymmetries critical for analysis of model and cell membranes. Existing biophysical techniques have different sensitivities to the precise interleaflet lipid organization in a bilayer, that can be maximized by optimizing experimental conditions and data analysis protocols. Coupling the *in vitro* approaches with simulations of corresponding atomistic models holds great promise for refining these methodologies, fully defining the state of the experimental systems and uncovering the full potential of membrane asymmetry for shaping and regulating bilayer properties.

## Author contributions

MD: conceptualization, data curation, formal analysis, investigation, project administration, writing – original draft, writing – review & editing. FAH: formal analysis, methodology, writing – original draft, writing – review & editing.

## Conflicts of interest

The authors declare no conflicts of interest.

## Data Availability

The data corresponding to this article have been stored at https://zenodo.org/records/14626488, https://zenodo.org/records/14655810, and https://zenodo.org/records/14627064.
